# Effect of three intracanal medicaments used in pulp regeneration on the push-out bond strength of mineral trioxide aggregate and calcium-enriched mixture: An *in vitro* study

**DOI:** 10.34172/joddd.2022.007

**Published:** 2022-05-29

**Authors:** Negin Ghasemi, Hamidreza Yavari, Mohammad Samiei, Naser Asl Aminabadi, Fatemeh Dabbaghi Tabriz, Samra Taheri, Paria Davoudi

**Affiliations:** ^1^Dental and Periodontal Research Center, Department of Endodontics, Faculty of Dentistry, Tabriz University of Medical Sciences, Tabriz, Iran; ^2^Department of Pediatric Dentistry, Faculty of Dentistry, Tabriz University of Medical Sciences, Tabriz, Iran; ^3^Department of Operative Dentistry, Faculty of Dentistry, Tabriz University of Medical Sciences, Tabriz, Iran; ^4^Faculty of Dentistry, Tabriz University of Medical Sciences, Tabriz, Iran; ^5^Department of Endodontics, Faculty of Dentistry, Shahid Beheshti Medical University, Tehran, Iran

**Keywords:** Bond strength, CEM, Intracanal medicament, MTA

## Abstract

**Background.** The bond strength of the materials used as a cervical barrier in the pulp regeneration is essential for the success of treatment. This study aimed to evaluate the effects of triple antibiotic paste (TAP), double antibiotic paste (DAP), and simvastatin as intracanal medicaments on the dislodgement resistance of mineral trioxide aggregate (MTA) and calcium-enriched mixture (CEM).

**Methods.** A total of 160 extracted human single-rooted teeth were selected, and root canal preparation was carried out. The teeth in each group were randomly divided into four subgroups: TAP, DAP, simvastatin, and the control group (without intracanal medicament). Four weeks after placing the medicaments, it was removed by sodium hypochlorite, and MTA and CEM were placed in the coronal third of the root canals. After a week, 2-mm-thick dentin disks were prepared from the coronal third of the roots, and the push-out test was performed using a universal testing machine. The data were analyzed using two-way ANOVA and independent *t*-test at a significance level of 0.05.

**Results.** Regardless of the intracanal medicament, there was no significant difference between the overall bond strength of MTA (59.3±10 MPa) and CEM (55.8±11 MPa) (*P*=0.6). Furthermore, there were no significant differences in bond strength between the two intracanal medicament groups and the control group (*P*>0.05).

**Conclusion.** Under the limitations of the current study, DAP, simvastatin, and TAP, as intracanal medicaments, did not adversely affect the push-out bond strength of CEM and MTA.

## Introduction

 The treatment of immature teeth with necrotic pulp with an open apex is one of the challenges in the endodontic field. Pulp regeneration is one of the methods used to treat these teeth to restore the physiological function of the tooth, considering the resulting continued longitudinal development of the root, the apical closure, and the increase in the thickness of the dentinal walls.^1,2^

 One of the most important steps in the regeneration treatment is root canal disinfection. Compared to mature teeth, these teeth cannot be cleaned and shaped like adult teeth due to their open apex and thin dentinal walls. The antimicrobial process relies more on intracanal medicaments and irrigation.^3^ In this context, the mixture of three antibiotics consisting of metronidazole, minocycline, and ciprofloxacin, known as the triple antibiotic paste (TAP), has acceptable root canal disinfection efficacy.^4-6^ The double antibiotic paste (DAP) is obtained by removing minocycline.^7^

 Simvastatin is an antimicrobial compound^8,9^ that has recently been considered in the endodontic field. This medicament belongs to the statin category and is used routinely to reduce blood fat. In addition to the therapeutic properties, this compound has the following properties^10-12^:

- Antimicrobial property against oral and periodontal pathogens, including bacteria, fungi, and viruses
- The potential to induce angiogenesis in pulp stem cells
- Affects the survival of osteoblasts and interferes with bone regeneration.

After root canal disinfection in the regeneration treatment, the next step is to fill the root canal with a resorbable matrix. Finally, a cervical barrier is placed in the coronal third of the root canal to prevent reinfection.^4^ Mineral trioxide aggregate (MTA) is a calcium silicate-based cement and is used as a cervical barrier. Its desirable properties include bioinductivity, biocompatibility, and the ability to induce pulp regeneration. Despite all its desirable characteristics, MTA has a long setting time; on the other hand, handling problems are always considered one of its disadvantages.^13,14^ Calcium-enriched mixture (CEM) is also an endodontic biomaterial with a calcium base that contains various calcium compounds. The clinical applications of this material are similar to those of MTA; however, it has a shorter setting time, better flow, and easier handling compared with MTA.^15,16^

 The sealing ability of materials used as cervical barriers in pulp regeneration is critical because bacterial leakage is a cause of the treatment failure, and a bacteria-free root canal is needed for successful regeneration.^17,18^ The bond strength of the materials to the root dentin is one of the factors indicating their sealing ability.^3^ These materials should be resistant to displacement forces caused by physiological function and the placement of restorative materials since their displacement will disrupt the sealing process and cause bacterial leakage.^19^ According to previous studies, the push-out test is a suitable method for evaluating the bond strength of materials.^13,20^

 The effect of intracanal antimicrobial materials such as TAP and DAP on the bond strength of the cervical barrier materials should be considered in the regeneration. Previous studies have shown that antibiotic pastes have acidic properties that cause dentinal erosion and collagen degeneration, and the mechanical bond of MTA affects the dentinal walls of the root canal.^1,3^ At present, two published studies are available on the effect of TAP and DAP on the bond strength of the ProRoot MTA.^1,3^ According to the results, they exert their negative effect on the bond strength of the MTA 4 weeks after being placed in the root canal. DAP decreases the bond strength compared with TAP,^1^ which has been rejected in another study, and there are contradictory reports in this regard.^3^

 The innovations of the current study, compared to the previous studies, are as follows:

1. Investigating the effect of DAP and TAP on CEM bond strength, which has not been studied by previous studies.
2. Evaluating the effect of simvastatin as an intracanal medicament on bond strength of MTA and CEM

This study aimed to investigate the effect of DAP, TAP, and simvastatin as intracanal medicaments on the push-out bond strength of MTA and CEM.

## Methods

###  Selection of teeth and preparation of the samples

 A total of 160 human teeth extracted for orthodontic reasons or periodontal disease were collected. The inclusion criteria for the study included:

1. Human tooth with a single root and a single canal [The single-canal characteristic was confirmed by taking radiographs from mesiodistal and buccolingual dimensions]
2. without previous root canal treatment
3. closed apex
4. no root caries
5. no calcification and erosion [Radiography examination]
6. no cracks and fractures in the root with visual examination and observation under an optical microscope at a magnification of ×40.

To equalize the length of the roots, the crown of each tooth was sectioned 2 mm above the cementoenamel junction using a diamond disk (SP 1600 Microtome, Leica, Nu Block, Germany) under water coolant so that all the roots measured about 14 mm [the crown of the teeth was not sliced completely so that temporary dressing can be placed during the study].

###  Root canal preparation and placement of intracanal medicaments

 The root canals were cleaned and shaped using the RaCe rotary system (FKG, Lachaux-de-Fonds, Switzerland) and the crown-down technique. The working length was determined by K-file #15 (Dentsply, Maillefer) so that when the file tip was observed at the apex, the final working length was obtained by subtracting 1 mm from this length. Master apical file (MAF) was set at #40 in all the specimens. To obtain the same standard diameter for each canal, Gates-Glidden drills (Dentsply Maillefer, Ballai­gues, Switzerland) were used, with 6, 5, 4, and 3 drills penetrating the root canal at depths of 3, 6, 9, and 12 mm, respectively. Sodium hypochlorite (2.5%) was used as an irrigation solution during instrumentation. To remove the smear layer at the end of the root canal preparation, 5 mL of 2.5% sodium hypochlorite and 17% EDTA were used for 1 minute. The final irrigation was performed using a normal saline solution.

 The specimens were divided into two groups (n = 80) based on the type of material used as a cervical barrier; group A: MTA, group B: CEM. Each group was divided into four subgroups based on the intracanal medicament: subgroup 1: TAP, subgroup 2: DAP, subgroup 3: simvastatin, and subgroup 4: control group (without intracanal medicament). These medicaments were placed using a lentulo spiral.

**TAP:** Equal proportions of metronidazole (Tehranshimi, Tehran, Iran), minocycline (Tehranshimi, Tehran, Iran), and ciprofloxacin (Ruzdarou, Tehran, Iran) were prepared as powder. These three powders were mixed with distilled water with a powder-to-liquid ratio of 3 to 1.

**DAP:** An equivalent combination of prepared ciprofloxacin and metronidazole powder was mixed with distilled water with a powder-to-fluid ratio of 2.5 to 1.

**Simvastatin:** 50 mg of the powder of this medicament, provided by the Pharmacy Co., was mixed with 1 mL of 100% ethanol.^21^

 After placing the intracanal medicaments up to the level of CEJ, the teeth were dressed with Cavit (ESPE, Norristown, PA). The specimens were incubated at 37°C under 100% moisture for four weeks. The root canal was empty in the control group and was dressed like the previous groups. After this period, the temporary dressing of the teeth was removed, and the antimicrobial materials were removed by 10 mL of 2.5% sodium hypochlorite and normal saline solution. The canals were dried with paper points.

###  Placement of cervical barriers and evaluation of the bond strength 

 The biomaterials used in the study were mixed according to the manufacturer’s instructions:

 MTA (Angelus, Londrina, Brazil): A powder-to-fluid ratio of 3:1 and hand mixing

 CEM (BioniqueDent, Tehran, Iran): Mixing the fluid with powder until achieving a thick consistency according to the manufacturer’s instructions

 MTA (A Group) and CEM (B Group) were transferred to the root canals by an MTA carrier and packed with a condenser with a suitable size. Parallel digital radiography confirmed that at least 4 mm of the coronal canal below CEJ was filled with the materials. A wet cotton pellet was placed on the materials, and the teeth were dressed with glass-ionomer (Fuji II Lc; GC Corp.; Tokyo, Japan).

 The specimens were incubated at 37°C for one week. To do the push-out test, the 2-mm discs were prepared from the coronal third of each root. The push-out test was performed by a universal testing machine (Hounsfield Test Equi­pment, Model HSK-S, Surrey, UK), which applied force to the specimens in the apico-coronal direction with a crosshead speed of 1 mm/min. The highest force that led to the bond failure was recorded in Newton (N).

 The push-out bond strength was calculated in MPa by dividing the recorded values in N by the surface area of the experimental specimen in mm^2^ calculated from the following formula:

 Surface area = 2πr × h (π = 3.14, r is the radius of the root canal, and h is the disk thickness in mm).

###  Statistical analysis

 After performing descriptive statistical analyses (means and standard deviations of the study data), the normal distribution of data was confirmed by the Kolmogorov-Smirnov test (*P* = 0.9). Two-way ANOVA was used to examine the significance of the effect of intracanal medicaments on the bond strength of the biomaterials, and the bond strength of CEM and MTA was compared using the independent t-test. P < 0.05 was considered the level of significance.

## Results

Table 1 presents the means and standard deviations of the study data. In both biomaterials, there were no significant differences between the bond strength of the subgroups treated with the intracanal medicaments and the control group (*P* > 0.05). The overall bond strengths in the MTA and CEM groups were not significantly different regardless of the intracanal medicament used (*P* = 0.6).

**Table 1:** Means ± SD of the bond strength for studied groups

**Medication**	**Group**	**Mean**	**SD**
TAP	MTA	56.6500	9.43637
	CEM	56.3000	13.59518
	Total	56.4750	11.39131
DAP	MTA	47.2800	9.08647
	CEM	53.9600	15.00572
	Total	50.6200	12.55039
Simvastatin	MTA	58.7000	14.98095
	CEM	62.2700	14.80338
	Total	60.4850	14.61046

## Discussion

 This study evaluated the effects of simvastatin, DAP, and TAP as intracanal medicaments on the displacement resistance of CEM and MTA as cervical barriers. The results indicated no adverse effects of these three compounds on the bond strength of the biomaterials.

 The pulp regeneration as an endodontic treatment for the root of immature teeth with necrotic pulp is based on the use of intracanal medicaments instead of conventional root canal cleaning and shaping.^18^ Another important treatment step is the placement of a biomaterial as a cervical barrier to activate the sealing property to prevent leakage of bacteria and their products, which are considered the most important factors for treatment failure.^3^ These two factors, i.e., placing the intracanal medicament and the cervical barrier (biomaterials), were considered two independent variables of this study. The dependent variable of this study was the displacement resistance of the material placed in the cervical region of the root, which indirectly indicates the sealing ability of the material and is measured using the push-out test.

 MTA and CEM are two endodontic biomaterials with a calcium silicate base used for various purposes such as regeneration of permanent immature teeth.^22^ Considering their physicochemical properties, these two materials were used as a cervical barrier in the present study. According to the results of the present study, regardless of the use of the intracanal medicament, the displacement resistance of these two materials was not significantly different, which is consistent with the results of studies conducted by Rahimi et al^13^ and Lotfi et al.^14^ Also, Yavari et al^23^ showed that the salivary microleakage was not significantly different in these two materials, used as a cervical barrier.

 Considering the release of calcium hydroxide during the setting reaction, both materials have the potential for chemical bonding with dentin because the released calcium hydroxide in combination with interstitial liquid phosphate forms a hydroxyapatite layer that forms the interstitial layer in combination with dentinal hydroxyapatite. This layer is responsible for the chemical bonding with the dentin.^24^ It has been shown in previous studies on MTA that DAP and TAP lead to collagen regeneration, and considering the destruction of the scaffold, they probably have a negative effect on displacement resistance;^1,17^ however, we did not obtain such a result, and there was no difference between the intracanal medicament-treated groups and the control group in terms of the bond strength to dentin.

 Simvastatin was also another compound used as an intracanal medicament in the present study. This medicament belongs to the statin family. This material has previously been used in various studies in the regeneration field. These studies have shown that it inhibits the production of inflammatory cytokines and increases the regeneration induction ability of the pulp cells and dentin formation. It also increases the activity of alkaline phosphatase.^10,11,25-27^ In order to be used as an intracanal medicament, a medicament must have a suitable antimicrobial property. Previous studies indicate the inhibitory effect of simvastatin on the biofilm of *Candida albicans* and *Staphylococcus aureus* and other species such as S*treptococcus sanguis, S. mutans, S. salivarius, S. sanguinis*, and *Porphyromonas gingivalis.*^12,28^ It did not have any adverse effect on the bond strength of the biomaterials studied. It also seems that after investigating its other properties, such as lack of any adverse effects on the physical properties of dentin and proper effect on *Enterococcus faecalis*, as one of the most resistant intracanal bacteria, it can be used as an intracanal medicament in regeneration processes. The simvastatin + TAP combination has also been evaluated in some studies under the title of 3 mixtatin^28,29^ and can be evaluated as an intracanal medicament in other studies.

 Attempts were made in the present study to minimize the effect of confounding factors on the push-out test. The specimens were kept under identical temperature and moisture. Materials used as a cervical barrier were placed and packed in one region by one person. Also, after cutting the discs into the same thickness of 2 mm, attempts were made to ensure that the materials were fully set in the disks. The diameter of the disks and the universal machine rod were 1.3 and 1.1 mm, respectively, and this ratio of diameters (90%) led to the least confounding effect on the reported bond strength.^20^ Attempts were made to ensure that the rod was perpendicular to the specimens and did not collide with the root canal walls during force application. The discs were checked for cracks after cutting. The cracked specimens and those that had not been tested were removed from the specimens and replaced by suitable specimens. Before placing the materials, the smear layer was removed to reconstruct the conditions of the clinical work. The intracanal medicaments were placed within the root canal for three weeks to comply with the guidelines recommended for clinical practice. Glass-ionomer was the temporary dressing used after the insertion of cervical materials instead of Cavit because Cavit absorbs the moisture of the area, interfering with the setting reaction of the hydration-based hydrophilic biomaterials.

 The numerical range obtained in the present study was similar to those reported by Topçuoğlu et al^3^ for MTA using calcium hydroxide, TAP, and DAP. The difference between the present study and the above study was that simvastatin and CEM were used in the present study. In addition, none of the previous studies and the present study did take into account the adverse effects of blood contamination,^13^ and this should be evaluated in future studies.

## Conclusion

 Under the limitations of the present study and according to the results of previous studies that have highlighted the properties of CEM, it can be concluded that this material, like MTA, can be used as a cervical barrier in the regeneration process. Considering its acceptable characteristics shown in previous studies, simvastatin can also be regarded as an intracanal medicament, provided that it exhibits other required characteristics in further studies.

## Acknowledgment

 This study was supported by a grant from Tabriz University of Medical Sciences. The authors would like to thank the Dental and Periodontal Research Center of Tabriz University of Medical Sciences.

## Authors’ Contribution

 Study concept and design by NG; study supervision by MS; critical revision of the manuscript for important intellectual content by NAA; statistical analysis by FDT; acquisition of data by ST; and drafting of the manuscript by NG and PD.

## Funding

 This study was supported by a grant from Tabriz University of Medical Sciences.

## Ethics Approval

 This study was approved by the Ethics Committee of Tabriz University of Medical Sciences (ethics code: IR.TBZMED.REC.1396.1056).

## Competing Interests

 There are no competing interests.
